# Perspectives in immunotherapy: meeting report from the “Immunotherapy Bridge 2018” (28–29 November, 2018, Naples, Italy)

**DOI:** 10.1186/s40425-019-0798-3

**Published:** 2019-11-29

**Authors:** Paolo A. Ascierto, Carlo Bifulco, Luigi Buonaguro, Leisha A. Emens, Robert L. Ferris, Bernard A. Fox, Greg M. Delgoffe, Jérôme Galon, Cesare Gridelli, Marco Merlano, Paul Nathan, Kunle Odunsi, Hideho Okada, Chrystal M. Paulos, Sandro Pignata, Kurt A. Schalper, Stefani Spranger, Giampaolo Tortora, Hassane Zarour, Lisa H. Butterfield, Igor Puzanov

**Affiliations:** 1 Unit of Medical Oncology and Innovative Therapy, Istituto Nazionale Tumori IRCCS Fondazione G. Pascale, Via Mariano Semmola, 80131 Naples, Italy; 2 Earle A. Chiles Research Institute, Robert W. Franz Cancer Research Center, Providence Portland Medical Center, Portland, OR USA; 3 Cancer Immunoregulation Unit, Istituto Nazionale Tumori IRCCS Fondazione G. Pascale, Naples, Italy; 4 UPMC Hillman Cancer Center, University of Pittsburgh, Pittsburgh, PA USA; 5 UPMC Hillman Cancer Center, University of Pittsburgh, Pittsburgh, PA USA; 6 Laboratory of Molecular and Tumor Immunology, Robert W. Franz Cancer Center in the Earle A. Chiles Research Institute at Providence Cancer Institute, Portland, Oregon, USA; 7 Department of Immunology, University of Pittsburgh, Pittsburgh, PA USA; 8 National Institute of Health and Medical Research, INSERM, Cordeliers Research Center, Paris, France; 9 Unit of Medical Oncology, Hospital “San Giuseppe Moscati”, Avellino, Italy; 10 Oncology Department, ASO Santa Croce e Carle Cuneo, Cuneo, Italy; 11 Mount Vernon Cancer Centre, Northwood, Middlesex, UK; 12 Department of Gynaecologic Oncology, Executive Director, Center for Immunotherapy, Roswell Park Comprehensive Cancer Center, Buffalo, NY USA; 13 Department of Neurological Surgery, University of California San Francisco, Parker Institute for Cancer Immunotherapy, San Francisco, California, USA; 14 Department of Microbiology and Immunology Hollings Cancer Center, Medical University of South Carolina (MUSC), Charleston, SC USA; 15 Uro-Gynaecological Department, Istituto Nazionale Tumori Fondazione G. Pascale, IRCCS, Naples, Italy; 16 Department of Pathology, Yale School of Medicine, Translational Immuno-oncology Laboratory, Yale Cancer Center, Medical Oncology, Yale School of Medicine and Yale Cancer Center, New Haven, CT USA; 17 The Koch Institute for Integrative Cancer Research at MIT and Department of Biology, Massachusetts Institute of Technology, Cambridge, MA USA; 18 Medical Oncology, Fondazione Policlinico Universitario Gemelli, IRCCS, Rome, Italy; 19 Melanoma Program, University of Pittsburgh Cancer Institute, Pittsburgh, PA USA; 20 Parker Institute for Cancer Immunotherapy Research Center, UCSF, San Francisco, California, USA; 21 Department of Medicine, Roswell Park Comprehensive Cancer Center, Buffalo, New York, USA

**Keywords:** Immunotherapy, Checkpoint inhibitors, Combination therapy, Biomarkers, Tumor microenvironment

## Abstract

Immunotherapy is now widely established as a potent and effective treatment option across several types of cancer. However, there is increasing recognition that not all patients respond to immunotherapy, focusing attention on the immune contexture of the tumor microenvironment (TME), drivers of the immune response and mechanisms of tumor resistance to immunity. The development of novel immunotherapeutics and their use in combination with checkpoint inhibitors and other standard of care and novel treatment modalities is an area of particular attention across several tumor types, including melanoma, lung, ovarian, breast, pancreatic, renal, head and neck, brain and non-melanoma skin cancers. The 4th Immunotherapy Bridge meeting (28–29 November, 2018, Naples, Italy) focused on a wide range of evolving topics and trends in the field of cancer immunotherapy and key presentations from this meeting are summarised in this report.

## Introduction

Immunotherapy is now established as a potent and effective treatment option across several cancer types. However, there is an increased recognition that not all patients respond to immunotherapy, highlighting the importance of the immune contexture of the tumor microenvironment (TME) as a driver of the immune response and tumor resistance to immunity and stressing the need for the development of novel immunotherapeutics and for their use in combination with checkpoint inhibitors and other standard of care and novel treatment modalities. The 4th Immunotherapy Bridge meeting (28–29 November, 2018, Naples, Italy) was focused on evolving topics and trends in cancer immunotherapy and is summarised in this report.

### Evolving topics in cancer immunotherapy: tumor microenvironment

#### Reprogramming the tumor microenvironment and T-cells for immunotherapy of ovarian cancer

Immune checkpoint inhibitors demonstrate promising but modest results in ovarian cancer (Table 1).

**Table 1.**
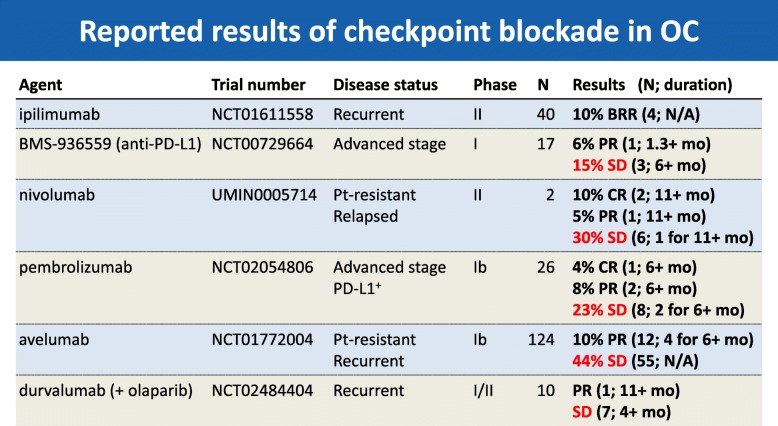
Reported results of checkpoint blockade in ovarian cancer.

In the KEYNOTE-100 trial, an overall response rate (ORR) of 8% was reported in patients with advanced recurrent ovarian cancer treated with pembrolizumab, with 29% of patients having stable disease [1]. PD-1 pathway blockade is only of limited benefit in ovarian cancer because of multiple immune suppressive networks in the TME. The challenge is how to increase antitumor T cell frequency and function through reprogramming the TME and promoting the persistence of anti-tumor T cells. One strategy is to utilize the cell destructive properties of oncolytic viruses. For instance, intratumoral administration of talimogene laherparepvec (T-VEC) plus pembrolizumab has been shown to increase CD8 infiltration and resulted in a 62% ORR in melanoma [2]. Different classes of oncolytic virus are currently being examined in ovarian cancer, including antigen-armed approaches. One of these is *Poxviridae* armed with a CXCR4 inhibitor. The CXCR4 receptor is one of the key stimuli involved in signaling interactions between tumor cells and their stromal microenvironment and is pivotal for metastasis and immune suppression within the ovarian TME. CXCR4 overexpression is related to an aggressive phenotype and poor prognosis in ovarian cancer and is essential for cancer-initiating cell maintenance, dissemination and metastatic spread to organs where CXCL12 is expressed. In an orthotopic ID8-T tumor model, a CXCR4 antagonist-expressing oncolytic vaccinia virus (OVV-CXCR4-Fc) led to reduced metastatic spread of tumors and improved overall survival (OS) compared with oncolysis alone. Inhibition of tumor growth was associated with reduced recruitment of T regulatory cells (Tregs), and higher ratios of interferon (IFN)-γ/interleukin (IL)-10+ tumor-infiltrating lymphocytes (TILs), as well as induction of spontaneous humoral and cellular antitumor responses [3]. Another strategy may be to use adoptive cell transfer (ACT) to render T cells resistant to immunosuppression by transforming growth factor (TGF)-β in order to promote persistence. The safety and feasibility of ACT has been established and a trial of NY-ESO-1 T-cell receptor (TCR) in ovarian cancer patients which offered evidence of adaptive immune resistance [4]. However, poor persistence may limit its use. Intrinsic TGFβ signaling blockade enhances in vivo persistence and a phase I/IIa study of TGFß blockade in TCR-engineered T cell cancer immunotherapy is now being assessed in patients with advanced malignancies.

## Key points

- PD-1 pathway blockade is only of limited benefit in ovarian cancer because of multiple immune suppressive networks in the TME.
- Different classes of oncolytic virus are currently being evaluated in ovarian cancer, including *Poxviridae* armed with a CXCR4 inhibitor and a CXCR4 antagonist-expressing oncolytic vaccinia virus (OVV-CXCR4-Fc).
- Another strategy may be to use ACT to render T cells resistant to immunosuppression by TGF-β in order to promote persistence.
- A phase I/IIa study of TGFß blockade in TCR-engineered T cell cancer immunotherapy is being conducted in patients with advanced malignancies.

### The contribution of tumor-residing dendritic cells to an anti-tumor immune response

CD8+ T cell inflammation is associated with an increased response to checkpoint blockade therapy. Tumor cell-intrinsic signaling pathways directly impact T cell infiltration into the TME. Molecular analysis of human metastatic melanoma samples revealed a correlation between activation of the WNT/β-catenin signalling pathway and absence of a T-cell gene expression signature [5]. Using a mouse melanoma model, a mechanism by which tumor-intrinsic active β-catenin signalling resulted in T-cell exclusion and resistance to anti-PD-L1/anti-cytotoxic T-lymphocyte-associated antigen (CTLA)-4 therapy was identified. Lack of CD103+ dendritic cells (DCs) was associated with reduced priming of tumor-specific T cells. Adoptive transfer of effector 2C T cells fails to control β-catenin-expressing tumors. T cells remain motile and migrate in a directional fashion after tumor eradication. However, β-catenin-expressing tumors show reduced tumor-reactive 2C T cell numbers with reduced motility. CD103+ dendritic cells are the predominant source of CXCR3 chemokine ligands and tumor-residing Batf3-driven CD103+ DCs are required for the recruitment of effector T cells into the TME as well as T cell priming in the tumor-draining lymph nodes [6]. Understanding the role of tumor-resident DCs may be important in improving response to immunotherapy. Regressing and progressing tumors exhibit differences in DC composition, with regressing tumors having higher numbers of cross-presenting DCs and CD8+ T cells. Regressing tumors mount T cell responses independent of CD103+ DC and conventional cross-presentation. Single cell RNA-sequencing has revealed new subsets of DCs associated with regressing tumors and thus associated with a highly productive anti-tumor immune response. A working hypothesis is that productive anti-tumor immunity depends on multiple tumor-resident DC subsets with cross-presenting capabilities.

## Key points

- Anti-tumor immune responses depend on priming and recruitment of CD8+ T cells.
- CD103+ cross-presenting DCs mediate priming and recruitment of CD8+ T cells into the TME.
- Tumor clearance is associated with prolonged functionality of cytotoxic T cells.
- New tumor-resident DC subsets have been identified associated with highly potent anti-tumor immunity.

### Understanding the immune composition and therapeutic implications of human lung cancer

**Fig. 1:**
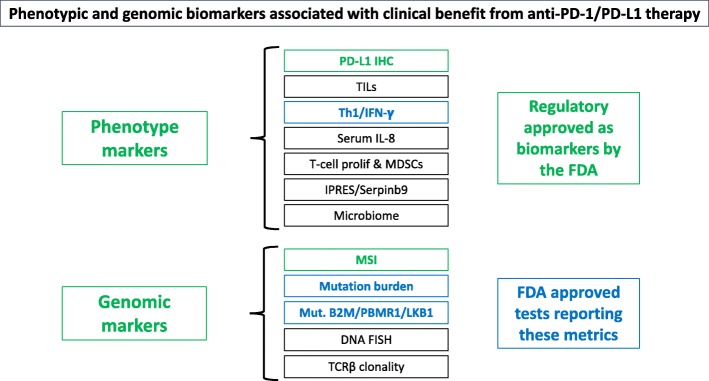
Phenotypic and genomic biomarkers associated with clinical benefit from anti-PD-1/PD-L1 therapy

The identification of predictive biomarkers is one of the major challenges in the field of immuno-oncology. Diverse biomarkers, including both phenotypic and genomic metrics, have shown association with benefit from PD-1/PD-L1 agents (Fig. 1).

However, the clinical use of these tests is limited by their suboptimal performance and limited understanding of their biological significance. To date, only elevated baseline PD-L1 and high microsatellite instability (MSI-H) have been approved for clinical use in multiple tumor types.

Emerging biomarkers such as tumor T-cell infiltration (or associated mRNA signatures) and increased tumor mutational burden may provide additional clinical value.

In previous studies using multiplexed and quantitative immunofluorescence analysis of major tumor-infiltrating lymphocyte (TIL) subpopulations, we have shown that increased levels of CD3 and CD8+ TILs are associated with better outcome in NSCLC, but only CD8 is independent from other prognostic variables [7].

Paired whole exome DNA sequencing and multiplexed quantitative immunofluorescence in pre-treatment samples from patients with NSCLC treated with PD-1 axis blockers revealed that elevated mutational load, candidate class-I neoantigens and intratumoral CD3 signal are significantly associated with favourable response to therapy [8]. Moreover, a ‘dormant’ TIL signature characterized by elevated TILs with low or moderate activation and proliferation was associated with a survival benefit in patients treated with immune checkpoint blockers. Dormant TILs were reinvigorated by PD-1 blockade in a patient-derived xenograft model. NSCLC can be stratified using T-cell markers into non-inflamed/poorly inflamed tumors (with low or virtual absence of CD3+ cells) and inflamed tumors with either low or moderate activation/proliferation (high CD3/low/modGZB and Ki-67) or high activation/proliferation (high CD3/high GZB or Ki-67). The presence of increased survival benefit in tumors with a “dormant” TIL phenotype than in “cold tumors” lacking TILs or in inflamed tumors with marked T-cell activation and proliferation indicate that effective immune stimulation using PD-1 axis blockers requires T-cells with specific functional profiles. Highly active/proliferating TILs may not be most sensitive to single-agent PD-1 blockade and this could be due, at least in part, to the common upregulation of multiple co-inhibitory signals in these cells.

PD-1, T cell immunoglobulin, mucin-3 (TIM-3) and lymphocyte activation gene 3 (LAG-3) are expressed in a proportion of NSCLCs with signals predominantly located in CD3+ T-cells [9].

These markers are positively associated with TILs and with each other; and negatively associated with KRAS and EGFR mutations in lung adenocarcinomas. In NSCLC patients with acquired resistance to PD-1 blocking agents, higher levels of TIL activation (granzyme B), proliferation (Ki-67), PD-1, TIM-3 and LAG-3 were associated with progression on-treatment [10]. Although multiple mechanisms may exist, up-regulation of immune inhibitory receptors such as TIM-3 and LAG-3 could mediate resistance to PD-1 axis blockers in a proportion of NSCLCs. Advanced analysis of the tumor immune contexture using a 29-marker imaging mass cytometry (IMC) panel showed increased CD4+/CD8+/CD20+ TILs with higher expression of functional markers in NSCLCs than case-matched non-tumor lung tissue [11]. Prominent differences in the T-cell profile were observed between patients with durable clinical benefit from immune checkpoint blockade compared with those without benefit, characterized by higher levels of effector memory CD8+/CD45RO+ TILs and lower levels of T-cell immune inhibitory receptors. Primary resistance to treatment was associated with CD4+ or CD8+ TILs containing increased levels of both activation (CD25/TBET/GZB/Ki-67) and immune suppression/dysfunction markers (PD-1/LAG-3/TIM-3/FOXP3). Taken together, these results suggest that prominent sensitivity to PD-1 axis blockers in NSCLC requires a defined tumor microenvironment characterized by the presence of TILs with a balanced activation/regulation profile. Expansion of these studies in larger cohorts and using computational multiparametric analysis is ongoing. Deep analysis of intact tumor specimens, circulating biomarkers and imaging and integration of data and computational analysis will be critical in identifying biomarkers that can be used to guide optimal immunotherapy.

## Key points

- Emerging biomarkers such as tumor T-cell infiltration (or associated mRNA signatures) and increased tumor mutational burden may be of clinical value.
- Sensitivity to PD-1 axis blockers in NSCLC requires a defined tumor microenvironment characterized by the presence of TILs with a balanced activation/regulation profile.
- Deep analysis of intact tumor specimens, circulating biomarkers and imaging and integration of data and computational analysis will be critical in identifying biomarkers that can be used to guide optimal immunotherapy.

### Fine-tuning T cell signal strength for optimal cancer immunotherapy

Although ACT is promising, how to improve the potency of TILs and chimeric antigen receptor (CAR) T cells for ACT is a critical issue. One solution may be to decrease the number of beads used in the TIL or CAR culture. Magnetic beads with CD3 and CD28 profoundly expand T cells, with three beads for one T cell the standard formula for CAR protocols. CD3/CD28 beads result in sustained logarithmic T cell growth, with T cells progressively differentiating into different effector T cells.

The use of 30-fold fewer Th17/CD3 ICOS beads per T cells still results in T cell growth and expansion. Moreover, T cell function is dramatically changed by using fewer beads, with T cells produced having greater functionality. A low signal strength induced polyfunctional cells, with a profound increase in cytokine production, including IL-17, IFN-γ, IL-22 and IL-2. T cells produced with fewer beads also had a less differentiated (‘younger’) phenotype. In a murine model, T cells produced with fewer beads resulted in a more effective antigen response. Low signal strength T cells also have a distinct metabolic profile characterized by reduced glycolytic activity and a higher spare respiratory capacity and oxidative phosphorylation.

Overexpression of phosphoenolpyruvate carboxy kinase 1 (PCK1) boosts the activity of murine CD4+ T cells, due to upregulation of phosphoenolpyruvate (PEP). Medium signal strength T cells express more PEP than high signal strength T cells. However, overexpressing PCK1 in high-stimulated CAR human Th17 cells can augments antitumor immunity. Thus, it may also be possible to engineer T cells with a modified metabolic profile leading to increased antitumor efficacy.

## Key points

- Improving the potency of TILs and CAR T cells for ACT is a critical issue.
- One option may be to decrease the number of beads used in the TIL or CAR culture, with T cells using fewer beads associated with greater functionality and a profound increase in cytokine production.
- It may be possible to engineer T cells with a modified metabolic profile leading to increased antitumor efficacy, such as through overexpression of PCK1 in high-stimulated CAR human Th17 cells.

### Overcoming metabolic barriers to effective antitumor immunity

The TME has an immunosuppressive landscape and engages in some very immunosuppressive functions. These include altering stromal cell function to support tumor growth, changing angiogenesis patterns, existing in multiple differentiation states, providing chronic antigen stimulation and recruitment of immunosuppressive cell types. However, a common phenotype of cancer is that it is hungry. Thus, the TME, driven by the metabolic derangement of tumor cells, generates a distinct metabolic landscape, involving hypoxia, lactic acidosis, hypoglycemia and essential amino acid depletion. An important question is whether the TMEs metabolic landscape presents a barrier to antitumor immunity and immunotherapy response. TIL are rendered metabolically insufficient and intratumoral T cells, especially CD8s, have striking metabolic defects. T cells infiltrating murine and human tumors demonstrate persistent loss of mitochondrial function and mass with repressed mitochondrial biogenesis causing T cell metabolic insufficiency [12]. However, enforcing mitochondrial biogenesis in tumor-specific T cells renders T cells resistant to metabolic insufficiency, which raises the question of whether metabolic support can be provided to T cells already in the TME.

Metabolic modulatory strategies to improve immunotherapies include genetic engineering approaches, the stimulation of programs that promote mitochondrial health, and pharmacological strategies to metabolically reprogram T cells (Table 2).

**Table 2.**
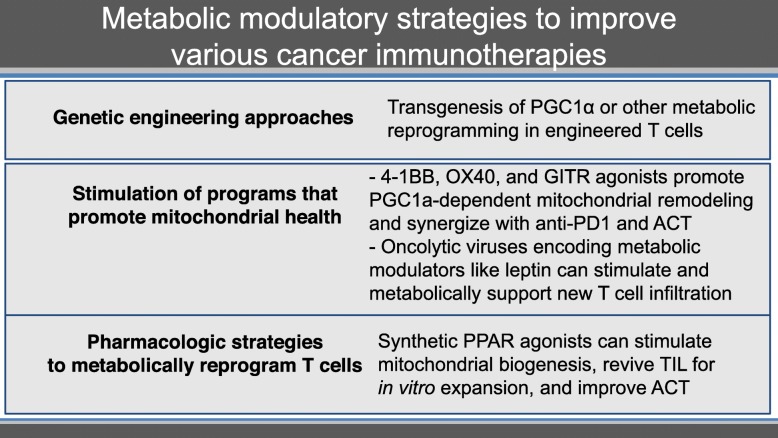
Metabolic modulatory strategies to improve various cancer immunotherapies.

However, the TME still presents metabolic barriers. Even if T cells are metabolically more competitive, they must still deal with the harsh conditions of the tumor. In addition, it is thought that there is vast metabolic heterogeneity in tumors, from tissue type, interpatient, and even between metastases of the same patient. Melanoma cell lines display considerable metabolic heterogeneity which can affect response to anti-PD-1 therapy. Oxidative, but not glycolytic, tumor cell metabolism prior to initiation of anti-PD-1 therapy was associated with a poor clinical outcome and, even in responding patients, low tumor oxygen consumption rates were associated with increased duration of response. Thus, tumor hypoxia is associated with resistance to PD-1 blockade. Therapeutic targeting of oxidative metabolism may be potentially beneficial. Metformin, a widely prescribed type 2 diabetes treatment, inhibited oxygen consumption in tumor cells in murine tumor lines resulting in reduced intratumoral hypoxia [13]. Combination of metformin with PD-1 blockade resulted in improved intratumoral T-cell function and tumor clearance. Metformin is now being assessed in combination with anti-PD-1 inhibitors in clinical trials in melanoma, squamous cell carcinoma of the head and neck (SCCHN) and colorectal cancer.

## Key points

- The TME generates a distinct metabolic landscape, which may present a barrier to antitumor immunity and immunotherapy response.
- Tumor hypoxia is associated with resistance to PD-1 blockade and targeting of oxidative metabolism may be potentially beneficial.
- Metformin, a widely prescribed type 2 diabetes treatment, inhibited oxygen consumption in tumor cells in murine tumor lines resulting in reduced intratumoral hypoxia and is now being assessed in combination with anti-PD-1 inhibitors in clinical trials.

### Immunotherapy in the neck: what’s new?

The treatment of patients with recurrent or metastatic locally advanced HNSCC is rapidly evolving. Cetuximab in combination with platinum and 5-fluorouracil still remains the standard of care as the first-line treatment. However, results of the CheckMate 141 trial appear to offer the first effective “second line” treatment after several years of failures. In this randomized phase III trial, patients with recurrent HNSCC whose disease had progressed within 6 months after platinum-based chemotherapy, nivolumab resulted in significantly longer OS compared with standard therapy (methotrexate, docetaxel, or cetuximab) [14]. However, only a small proportion of patients were responsive to nivolumab (13.3% versus 5.8% with standard therapy) and no predictive markers of response were identified. Similarly, first line treatment with pembrolizumab significantly improved OS compared with cetuximab plus carboplatin or cisplatin (EXTREME) in patients with increased PD-L1 expression and was non-inferior in the total population in the KEYNOTE-048 trial [15]. Pembrolizumab plus cisplatin or carboplatin significantly improved OS versus EXTREME in the total population. Pembrolizumab also had a favorable safety profile versus EXTREME and these data support pembrolizumab monotherapy as a new first-line standard of care for PD-L1+ recurrent HNSCC.

Several promising immunotherapy agents are also under development in head and neck cancer, including toll-like receptor (TLR)-agonists, αSTAT-3, αNKG2A, and αTGF-β. SD-101 is an agonist of TLR9 that stimulates DCs to release IFN-α and mature into antigen-presenting cells to activate T cell anti-tumor responses. In anti-PD-1 treatment-naïve recurrent and/or metastatic HNSCC patients, SD-101 in combination with pembrolizumab showed a promising response rate, appearing to enhance the systemic effect of anti-PD-1 blockade, and was well tolerated [16]. The TGF-β pathway promotes tumor immunosuppression and its inhibition may enhance the antitumor activity of PD-1/PD-L1 inhibitors. M7824 is a bifunctional fusion protein composed of an anti-PD-L1 fused with the extracellular domain of TGF-βRII. In a phase I trial, M7824 showed promising clinical activity (ORR of 22%) and a manageable safety profile in patients with refractory/metastatic HNSCC [17].

There was a possible trend toward higher activity in HPV + patients (ORR 50%) and evidence of clinical activity irrespective of PD-L1 status. In another study, danvatirsen, an antisense oligonucleotide STAT3 inhibitor, resulted in a higher response rate in combination with durvalumab versus durvalumab monotherapy in PD-L1 treatment-naïve patients with recurrent/metastatic-HNSCC [18].

Targeting new inhibitory receptors other than PD-(L)1 may also have a potential role. Monalizumab targets NKG2A receptors expressed on tumor-infiltrating cytotoxic NK and CD8 T lymphocytes. Preliminary data suggest promising antitumor activity of monalizumab in combination with cetuximab in patients with HNSCC progressing after platinum-based therapy with acceptable safety [19].

## Key points

- PD-1 inhibitors have shown promising results in patients with recurrent or metastatic locally advanced SCCHN and data support pembrolizumab monotherapy as a new first-line standard of care for PD-L1+ recurrent SCCHN.
- Several promising immunotherapy agents are under development in head and neck cancer, including TLR-agonists, αSTAT-3, αNKG2A, and αTGF-β.
- Targeting inhibitory receptors other than PD-(L)1 may also have a potential role; monalizumab targets NKG2A receptors expressed on tumor-infiltrating cytotoxic NK and CD8 T lymphocytes and preliminary data suggest promising antitumor activity in combination with cetuximab.

### Immunotherapy in GU: what’s new?

In the phase III CheckMate-214 trial, OS and ORR were significantly higher with nivolumab plus ipilimumab than with sunitinib among intermediate-risk and poor-risk patients with previously untreated advanced renal-cell carcinoma (RCC) [20]. 18-month OS rate was 75% with nivolumab plus ipilimumab and 60% with sunitinib. Treatment-related adverse events leading to discontinuation occurred in 22% of patients in the nivolumab plus ipilimumab group and 12% of patients in the sunitinib group. Nivolumab plus ipilimumab represents a new standard of care for intermediate- or poor-risk advanced RCC. In the IMmotion151 trial, atezolizumab was combined with bevacizumab and compared with sunitinib as first-line treatment in metastatic RCC. Median progression-free survival (PFS) was significantly longer with atezolizumab plus bevacizumab in patients with PD-L1 expression (≥1%) (11.2 versus 7.7 months with sunitinib) and tolerability was consistent with monotherapies [21]. Tumor molecular analyses showed that high T effector/IFN-γ (T_eff_) gene expression signature was associated with PD-L1 expression and longer PFS for atezolizumab plus bevacizumab compared to sunitinib [22]. Angiogenesis gene expression was higher in the favorable Memorial Sloan Kettering Cancer Center (MSKCC) risk group but lower in sarcomatoid tumors, in which PD-L1 expression was higher. The differential activity of atezolizumab plus bevacizumab between tumors with angiogenic and immunogenic phenotypes is not robust enough for clinical decision-making.

Axitinib, a more selective and potentially less toxic vascular endothelial growth factor (VEGF) inhibitor, was combined with pembrolizumab in a phase 1b study in patients with treatment-naive advanced RCC. The combination was tolerable and showed promising antitumor activity [23].

The combination has also shown significantly improved OS and PFS versus sunitinib as first-line therapy for advanced or metastatic RCC in the KEYNOTE-426 trial [24]. Axitinib has also been evaluated in combination with avelumab in the JAVELIN renal 100 trial, with manageable toxicity and encouraging antitumor activity in preliminary analysis [25]. The combination significantly improved PFS in patients with PD-L1+ expression, with PFS and ORR benefits also observed in patients irrespective of PD-L1 expression and across all prognostic risk groups [26].

Tyrosine kinase inhibitor plus immunotherapy combinations have shown an efficacy signal in all risk groups but have not been compared with ipilimumab plus nivolumab. Axitinib plus pembrolizumab data are awaited to assess whether there is any advantage of anti-PD-1 versus anti-PD-L1 in RCC. Another consideration moving forward is that heterogeneity is particularly marked in RCC. Tumor mutational burden (TMB) is modest with no correlation with activity of atezoluzimab plus bevacizumab. RCC has the highest pan-cancer proportion and number of indel mutations, with evidence suggesting these are a highly immunogenic mutational class which can trigger an increased abundance of neoantigens [27]. Identification of truncal neo-antigens may provide a target for cellular therapies.

## Key points

- Combination immunotherapy with ipilimumab plus nivolumab represents a new standard of care for intermediate and poor risk metastatic RCC patients
- Combinations of anti-PD1 or PDL1 antibodies with anti-VEGF agents have shown superiority to anti-VEGF agents alone and will become an option for all prognostic groups of patients with metastatic RCC.
- PDL-1 expression is not an adequate biomarker in RCC to direct therapeutic decisions.

### Immunotherapy for ovarian cancer. How to move forward

Multiple clinical studies demonstrate a correlation between TILs and survival in ovarian cancer, independent of tumor grade, stage or histologic subtype [28]. PD-1/PD-L1 inhibitors have demonstrated encouraging but modest activity in recurrent ovarian cancer, suggesting an opportunity for combinations. In KEYNOTE-100, pembrolizumab was associated with antitumor activity in patients with recurrent advanced ovarian cancer with 1–2 or 3–5 prior lines of therapy, with ORR increasing with PD-L1 expression [1]. The anti PD-L1 agent avelumab is also being tested in two ongoing trials in ovarian cancer. In the JAVELIN OVARIAN 200 trial, patients with platinum-resistant/refractory disease are randomized to avelumab, pegylated liposomal doxorubicin or both combined, while in the JAVELIN OVARIAN 100 trial, previously untreated patients are randomized to carboplatin and paclitaxel with or without avelumab before a maintenance period in which patients in the avelumab arm continue on therapy while patients who received platinum-based therapy without avelumab will be randomized to avelumab or observation.

Other strategies involve immunotherapy in combination. In the ENGOT-ov39 trial (IMagyn050), post-surgery patients will be randomized to carboplatin plus paclitaxel plus bevacizumab with or without atezolizumab with initial treatment followed by maintenance bevacizumab with or without atezolizumab until completion, toxicity or recurrence. Pre-clinical data have also suggested synergy between anti-PD-1 therapy and poly-ADP ribose polymerase (PARP) inhibition. PARP inhibitors up-regulate PD-L1 expression in preclinical models which could potentiate an anti-tumor immune response. Niraparib is an oral PARP inhibitor approved for maintenance treatment of recurrent ovarian cancer. In a phase I/II study of patents with platinum-refractory ovarian cancer, ORR was 25% and disease control rate was 68% among 60 evaluable patients [29]. In 12 patients with BRCA-mutated tumors, the ORR was 45%. Similar ORRs were achieved irrespective of homologous recombination deficiency (HRD) and BRCA status in the platinum-resistant/refractory subgroup. Several phase III trials involving over 4000 patients are ongoing or planned to assess the combination of anti-PD-1/PD-L1 therapy with a PARP inhibitor.

## Key points

- PD-1/PD-L1 inhibitors have demonstrated encouraging but modest activity in recurrent ovarian cancer, suggesting an opportunity for combinations.
- Pre-clinical data have suggested synergy between anti-PD-1 therapy and PARP inhibition, with PARP inhibitors up-regulating PD-L1 expression in preclinical models.
- Several phase III trials are ongoing or planned to assess the combination of anti-PD-1/PD-L1 therapy with a PARP inhibitor.

### Immunotherapy: turning up the heat on breast cancer

Of the breast cancer subtypes, triple-negative breast cancer (TNBC) is a particularly attractive candidate for cancer immunotherapy. Median OS is 9–18 months in the metastatic setting and there are few current targeted therapy options. TNBC also has a higher rate of mutational complexity and PD-L1 expression and is more likely to harbor TILs.

In a phase I study, women with metastatic TNBC received atezolizumab every 3 weeks until unacceptable toxic effects or loss of clinical benefit [30]. Prior to receiving atezolizumab, most patients were heavily pretreated. Single agent atezolizumab was well-tolerated and clinically active (Table 3).

**Table 3.**
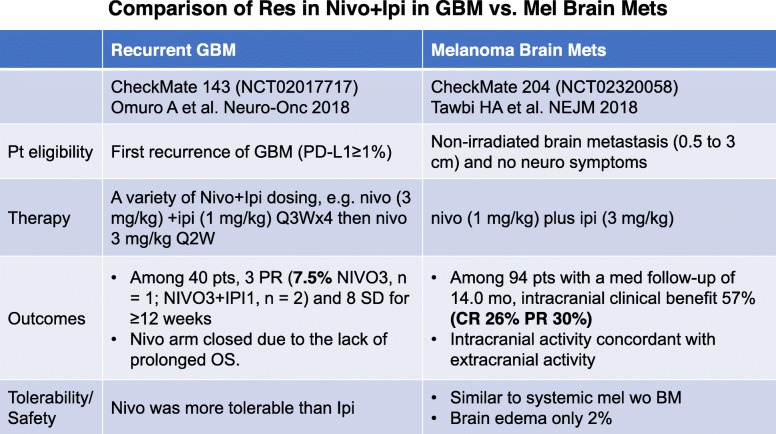
Clinical Activity Associated with Atezolizumab Monotherapy in the Phase 1 PCD48989g Study

Median PFS was 1.4 months by RECIST, and 1.9 months by irRC; objective response rates by RECIST and irRC were 10 and 13%. Clinical benefit was durable, with a median duration of response (DOR) of 21 months by RECIST, and 25 months by irRC. Exploratory analyses identified line of therapy for advanced disease and immune biomarkers as factors that may predict clinical benefit (Table 3).

While the median OS in all patients was 8.9 months, in first-line patients it was 17.6 months. Patients with PD-L1 expressing tumor-infiltrating immune cells in ≥1% of the tumor area had higher ORRs and longer OS. Levels of tumor-infiltrating immune cells > 10% was also independently associated with higher ORRs and longer OS. Clinical benefit was observed in some patients with RECIST v1.1 stable or progressive disease.

Molecular characterization of atezolizumab-treated patients showed a median TMB of 4.6 Mut/Mb [31]. TMB was not associated with either TILs or immune biomarkers, or with clinical activity (ORR, PFS or OS). Loss of heterozygosity, mutations in TP53, or mutations in BRCA1/2 were not associated with clinical response to atezolizumab. Clinical benefit from atezolizumab was enriched in basal-like immune-activated (BLIA) and luminal androgen receptor (LAR) TNBC subtypes, both of which indicate tumors with a more active tumor immune microenvironment. Higher antigen presentation and T_eff_ gene expression signatures were also associated with increased clinical activity.

Standard cancer therapies can augment the activity of immunotherapies and the combination of PD-1/PD-L1 blockade with standard chemotherapy is being evaluated in TNBC. In the IMpassion 130 study, patients with untreated metastatic TNBC were randomized to atezolizumab plus nab-paclitaxel or placebo plus nab-paclitaxel until disease progression or unacceptable toxicity [32]. The combination was generally safe and well-tolerated; adverse events that led to the discontinuation of any agent occurred in 15.9% of the patients who received atezolizumab plus nab-paclitaxel and in 8.2% of those who received nab-paclitaxel monotherapy. Median PFS was 7.2 months with atezolizumab plus nab-paclitaxel compared with 5.5 months with nab-paclitaxel alone, while median OS was 21.3 versus 17.6 months. In patients with PD-L1+ tumors, median PFS was 7.5 and 5.0 months and median OS was 25.0 and 15.5 months, respectively. Based on these data, atezolizumab and nab-paclitaxel received accelerated approval by the FDA. The future is in combination immunotherapies that both promote the induction of active T cells and relieve immune suppression. These strategies should have synergistic clinical activity, though could also result in increased toxicity.

## Key points

- Triple negative breast cancers (TNBC) are more likely to harbor TILs and express PD-L1 than other breast cancers.
- Atezolizumab monotherapy is well tolerated and has durable clinical activity in some patients with metastatic TNBC, with response rates of 10–13% overall.
- The clinical activity of single agent atezolizumab is higher when used in the first-line setting for advanced disease, and in patients who are > PD-L1 IC positive, with response rates of 24% and 12–16% respectively.
- A randomized Phase 3 clinical trial of atezolizumab with nab-paclitaxel versus placebo with nab-paclitaxel in TNBC patients with untreated metastatic disease demonstrated that adding atezolizumab to nab-paclitaxel is safe and feasible, with response rates of 56% in all patients and 59% in > PD-L1 IC+ patients.
- In untreated PD-L1 IC+ TNBC patients, the atezolizumab/nab-paclitaxel combination resulted in a PFS benefit of 2.5 months, and an OS benefit of 9.5 months.

### Immunotherapy in pancreatic cancer: lights and shadows

Studies of single-agent immunotherapy in pancreatic ductal adenocarcinoma (PDAC) have been disappointing with PD-1/PD-L1 checkpoint inhibitors largely ineffective. Activity of pembrolizumab has been shown only in mismatch repair (MMR) deficient tumors, which represent only around 1% of PDAC cases [33]. PDAC is considered as a non-immunogenic, or cold, tumor type with many mutations but very few neoantigens. PDAC offers a strongly immune resistant and suppressive environment. Lack of response may in part be due to PDAC’s unique TME, consisting of a dense fibrotic stroma and a scarcity of TILs. However, it is not the physical barrier of the stroma but rather an oncogene-driven immunosuppressive network that excludes effector T cells. In reality, nearly all PDAC samples harbor potentially targetable neoantigens. In fact, T cells are present but generally show a reduced activation signature and markers of antigen presentation are associated with a reduced signature of markers characterizing cytotoxic T cells [34]. These findings suggest that despite the presence of tumor specific neoepitopes, T cell activation is actively suppressed in PDAC. Interestingly, contrary to other tumors, mutation load in PDAC is inversely related with T-cell activity.

Chemokines and their receptors play a critical role in conditioning metastatic niche, immunosuppressive status and the TME. They help to recruit to tumor side and to ‘corrupt’ neutrophils, monocytes/macrophages and fibroblasts with different properties which, together, help tumor growth and metastatic spread. CXCR2 signaling is upregulated in myeloid-derived suppressor cells and in pancreatic cancer and CXCR2 inhibition in mice enhances T cell entry and confers sensitivity to anti-PD-1 therapy [35]. Another possible approach is targeting macrophages through CSF1R inhibitors. Macrophages functionally contribute to the squamous subtype of human PDAC and inhibition of CSF1R alters the TME and results in an enhanced T cell immune response [36].

Long-term survivors of PDAC display evidence of enhanced tumor-specific T-cell responses that are associated with unique neoepitope quality but not quantity [37]. Multiplexed immunohistochemistry revealed no difference in the absolute number of CD3+ T cells between long-term and short-term survivors. However, there was a threefold increase in cytotoxic CD8+ T cells, in long-term survivors and an increase in the number of cytolytic CD8+ T cells, including CD3+, CD8+, and granzyme B+ cells. Using whole-exome sequencing and in silico neoantigen prediction, tumors with both the highest neoantigen number and the most abundant CD8+ T-cell infiltrates, but neither alone, stratified patients with the longest survival. Long-term survivors displayed persistent T cell clones that cross-react with tumor neoepitopes and homologous microbial antigens; the theory of molecular mimicry postulates that T cell receptors that can recognize pathogenic antigens can also recognize non-pathogenic antigens. This could guide the selection of patients for immuno-oncology treatment protocols and for the design of individualized peptide-based vaccines, selecting peptides that are predicted by this computation of neoepitope quality to be the most likely to generate an effective immune response.

Priming or boosting of T cell responses is required for therapeutic effect and sensitization to checkpoint blockade in PDAC. Most tumors are unresponsive to immune checkpoint blockade, especially if deep immunosuppression in the tumor develops prior to and prevents T cell immunosurveillance. Failed or frustrated T cell priming often needs repair before successful sensitization to PD-1/PD-L1 blockade. Large numbers of clinical trials of checkpoint inhibitors combined with other agents are planned or ongoing in an effort to achieve this goal.

## Key points

- PDAC offers a strongly immune resistant and suppressive environment. and studies of single-agent immunotherapy in PDAC have been disappointing with PD-1/PD-L1 checkpoint inhibitors largely ineffective.
- Despite the presence of tumor specific neoepitopes, T cell activation is actively suppressed in PDAC, and contrary to other tumors, mutation load in PDAC is inversely related with T-cell activity.
- Priming or boosting of T cell responses is required for therapeutic effect and sensitization to checkpoint blockade in PDAC and large numbers of clinical trials of checkpoint inhibitors combined with other agents are planned or ongoing in an effort to achieve this goal.

### Immunotherapy for brain cancer

In patients with melanoma brain metastases, nivolumab plus ipilimumab resulted in an intracranial clinical benefit of 57% (26% complete responses) with intracranial activity concordant with extracranial activity [38]. However, in patients with recurrent glioblastoma, treatment with nivolumab with or without ipilimumab resulted in only three of 40 patients achieving a partial response and eight having stable disease for ≥12 weeks [39] (Table 4).

**Table 4.**
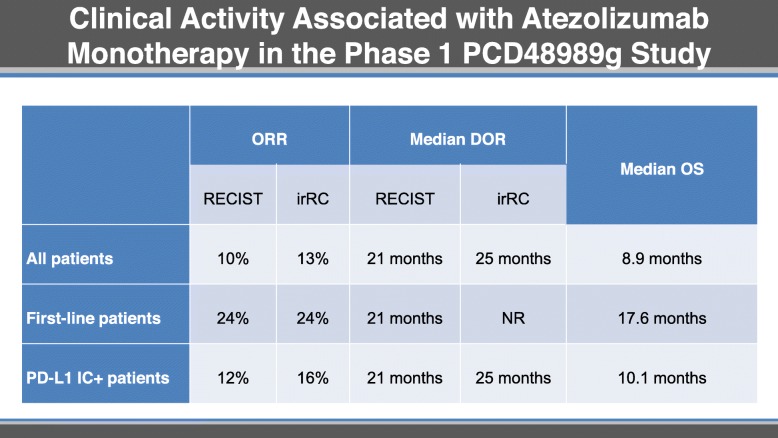
Comparison of nivolumab plus ipilimumab in glioblastoma and melanoma brain metastases

Nivolumab monotherapy was better tolerated than nivolumab plus ipilimumab but the monotherapy arm was closed early due to poor OS. Compared with melanoma brain metastases, glioblastoma is more infiltrative, and thus more protected by the blood-brain barrier with penetration of IgG antibodies of only around 4%. Recurrent glioblastoma may have more profound lymphopenia. Moreover, melanomas have more mutations, thereby more neoantigens. Gliobastoma is considered a cold tumor medium with a low TMB, although chemotherapy with temozolomide often induces hypermutation. However, whether cold tumor status is solely attributable to the low mutation load is unclear. Case reports on durable responses to immune checkpoint inhibition in hypermutant glioblastoma arising from primary genetic predisposition or secondary MMR deficiency suggest yes, whereas other data indicate tumor mutational load was not associated with CD8+ T cell infiltration or PD-1/PD-L1 expression based on evaluation of 198 glioblastoma cases [40].

There is evidence to suggest the brain is surprisingly susceptible to adoptively transferred T-cells. T cell receptor-targeting MAGE-A3 has been reported to cause severe damage to brain grey matter, resulting in two deaths. High IL-6, IL-2, granulocyte-macrophage colony-stimulating factor (GM-CSF) and VEGF levels in cerebrospinal fluid (CSF) have been observed during neurotoxicity, with both CD20 CAR and non-CAR T cells accumulating in the CSF and in the brain parenchyma. There is also evidence of endothelial activation, disseminated intravascular coagulation, capillary leak, and increased blood-brain barrier permeability in the CD19-CAR setting. A single dose of peripherally infused EGFRvIII-directed CAR T cells mediated antigen loss and induced adaptive resistance in patients with recurrent glioblastoma [41]. In situ evaluation of the tumor environment demonstrated increased and robust expression of inhibitory molecules and infiltration by regulatory T cells after CART-EGFRvIII infusion. Overcoming adaptive changes in the local TME and addressing antigen heterogeneity may improve the efficacy of EGFRvIII-directed strategies in glioblastoma.

## Key points

- Anti-PD-1 agents have shown efficacy in patients with melanoma brain metastases but less so in patients with recurrent glioblastoma, which is more protected by the blood-brain barrier and has with a lower TMB.
- There is evidence to suggest the brain is surprisingly susceptible to adoptively transferred T-cells. T cell receptor-targeting MAGE-A3 has been reported to cause severe damage to brain grey matter.
- Overcoming adaptive changes in the local TME and addressing antigen heterogeneity may improve the efficacy of EGFRvIII-directed strategies in glioblastoma.

### Immunotherapy evolution for lung carcinoma

Single-agent pembrolizumab is now the standard of care for advanced NSCLC with PD-L1 expression of ≥50% [42], although it is not superior to chemotherapy in NSCLC PD-L1 < 50%. Nivolumab is not superior to chemotherapy regardless of PD-L1 expression [43]. Results from two phase III randomized trials of atezolizumab versus chemotherapy are pending.

With regard to anti-PD-1/PD-L1 therapy in combination with chemotherapy, pembrolizumab plus pemetrexed and a platinum-based drug significantly prolonged OS versus chemotherapy alone in patients with metastatic non-squamous NSCLC [44]. Improved OS was seen across PD-L1 subgroups. The addition of pembrolizumab to chemotherapy of carboplatin plus paclitaxel or nab-paclitaxel also resulted in significantly longer OS and PFS than chemotherapy alone in patients with previously untreated metastatic, squamous NSCLC [45]. First-line treatment with nivolumab plus chemotherapy also improved PFS versus chemotherapy alone in patients with non-squamous NSCLC with PD-L1 expression < 1% [46]. Phase III randomized trials have failed to show OS superiority for atezolizumab plus chemotherapy compared with chemotherapy alone except for the combination of atezolizumab with carboplatin, paclitaxel and bevacizumab in non-squamous patients in the Impower 150 trial [47]. Atezolizumab plus chemotherapy improved PFS but failed to show a survival benefit as first-line treatment in stage IV squamous NSCLC [48].

Combined immunotherapy may also have a role in the treatment of NSCLC. However, the combination of tremelimumab plus durvalumab did not improve OS or PFS compared with chemotherapy in unselected patients with lung cancer [49]. In another phase III trial, PFS in NSCLC patients with a high TMB was significantly longer with nivolumab plus ipilimumab than with chemotherapy [50]. The benefit of nivolumab plus ipilimumab over chemotherapy was broadly consistent within subgroups, including patients with a PD-L1 expression level of ≥ or < 1%, although results seemed to be more impressive in PD-L1 < 1% patients. Recent doubts about the predictive role of TMB, with a survival gain even in low TMB patients, means nivolumab plus ipilimumab could offer a chemotherapy-free option for advanced NSCLC regardless of PD-L1 and TMB status.

## Key points

- Single-agent pembrolizumab is now the standard of care for advanced NSCLC with PD-L1 expression of ≥50%.
- Combined immunotherapy may also have a role in the treatment of NSCLC with PFS in NSCLC patients with a high TMB significantly longer with nivolumab plus ipilimumab than with chemotherapy.
- Recent doubts about the predictive role of TMB, with a survival gain even in low TMB patients, means nivolumab plus ipilimumab could offer a chemotherapy-free option for advanced NSCLC regardless of PD-L1 and TMB status.

### Immunotherapy for Merkel cell carcinoma: present and future

Merkel cell carcinoma (MCC) is a rare, aggressive skin cancer associated with poor survival. In a phase I trial, first-line therapy with pembrolizumab in patients with advanced MCC was associated with an ORR of 56%, with responses in patients with and without virus-positive tumors [51]. Subsequent data with a median follow-up of 8.6 months showed durable tumor control, a favorable OS rate of 68% at 18 months and a manageable safety profile [52]. Similar results have been observed with nivolumab, which induced rapid and durable tumor regressions in treatment-naive and previously-treated patients with advanced MCC (ORR 68%) in both virus-positive and virus-negative tumors [53].

In a phase II trial in patients with stage IV chemotherapy-refractory MCC, avelumab was associated with durable responses and an ORR of 31.8% [54]. Avelumab was also well tolerated with five grade 3 treatment-related adverse events occurring in four (5%) patients. In subsequent follow-up, 1-year PFS rate was 30% and 1-year OS rate was 52% [55]. Median OS was 12.9 months. Subgroup analyses suggested a higher probability of response in patients receiving fewer prior lines of systemic therapy, with a lower baseline disease burden, and with PD-L1+ tumors; however, durable responses occurred irrespective of baseline factors, including tumor Merkel cell polyomavirus status.

Avelumab has also achieved high rates of response and was well tolerated as first-line therapy in patients with distant metastatic MCC, with a confirmed ORR of 62.1% [56]. These data are supported by results of a European expanded access programme, which has reported physician-assessed objective responses in 54.3% (*n* = 57) of patients and a disease control rate (DCR) of 75% [57]. Taken together, these data suggest that checkpoint inhibitors may represent a new standard of care for advanced MCC.

PD-1/PD-L1 checkpoint inhibitors may also have a role as neo-adjuvant or adjuvant therapy. In the first trial of anti-PD-1 treatment in the neoadjuvant setting for resectable MCC, nivolumab administered for 4 weeks before surgery was safe and induced substantial radiologic and pathologic tumor regressions in 45 and 65% of patients, respectively [58]. Among 21 patients followed after surgery, all were progression-free at 6 months and two had relapsed at 12 months. Nivolumab and avelumab are also being assessed in adjuvant trials.

## Key points

- Durable tumor control and a manageable safety profile have been shown with pembrolizumab and nivolumab in patients with Merkel cell carcinoma.
- Avelumab was also associated with durable responses and was well tolerated in patients with stage IV chemotherapy-refractory MCC and in patients with distant metastatic MCC.
- Taken together, these data suggest that checkpoint inhibitors may represent a new standard of care for advanced MCC.
- PD-1/PD-L1 checkpoint inhibitors may also have a role as neo-adjuvant or adjuvant therapy.

### Drivers of immune responses

#### Metabolic properties of immune cells in renal cell carcinoma: opportunities for therapeutic optimization

Clear cell RCC has the highest CD8a signature of all non-lymphoid solid tumor types and RCC is often, but not always, immune responsive. PD-1 checkpoint inhibition can be effective, but only in around 25% of patients with clear cell RCC and duration of responses can be limited.

Clear cell RCC CD8 TILs have been shown to be phenotypically distinct and poorly functional [59]. CD8 TILs from clear cell RCC patients appear effector-memory-like and are PD-1^high^, which may indicate chronic stimulation and a suppressed or exhausted state. Clear cell RCC CD8 TILs also had a broad set of defects in glucose uptake, glycolysis, and mitochondrial dynamics and function, which contribute to their limited capacity to activate. RCC TILs fail to utilize glucose for stimulation, in spite of access to adequate glucose and appropriate expression of nutrient tools. T cell activation can be partially restored with pyruvate or mitochondrial reactive oxygen species (ROS) scavengers. Metabolic adaptations of TILs to the clear cell RCC TME may form a barrier to antitumor immunity and immune-checkpoint therapy.

Mitochondrial deficiencies appear to play key roles in the inability of TILs to eliminate cancer cells. Mitochondria in clear cell RCC CD8 TILs are abundant but appear small and highly fragmented compared with normal healthy CD8 T cell mitochondria, and are highly polarized with increased mitochondrial ROS. Tumor-specific CD8 T cells with mitochondrial hyperpolarization express inhibitory receptors and have limited ability to control tumors in studies of adoptive T cell therapy.

Metabolic defects contribute to poor T cell function in tumors. Immune therapies can, however, reactivate T cells to promote antitumor immunity, if only in a relatively small subset. It is important to understand mechanisms that may restrict T cell metabolism in clear cell RCC in order to provide new markers for T cell function and potential targets to improve T cell-mediated antitumor immunity.

## Key points

- PD-1 checkpoint inhibition can be effective in patients with clear cell RCC, but only in around 25% and duration of responses can be limited.
- Metabolic adaptations of TILs to the clear cell RCC TME may form a barrier to antitumor immunity and immune-checkpoint therapy.
- Mitochondria in clear cell RCC CD8 TILs are abundant but appear small and highly fragmented compared with normal healthy CD8 T cell mitochondria and are highly polarized with increased mitochondrial ROS.
- It is important to understand mechanisms that may restrict T cell metabolism in clear cell RCC in order to provide new markers for T cell function and potential targets to improve T cell-mediated antitumor immunity.

### Cancer vaccines and strategy to develop combination immunotherapy with T cell agonists

Advanced cancers tend to be heterogeneous so complete response will require immunity against a large number of antigens. As such, a goal of treatment should be to provide a large number of relevant antigens in order to activate a broad spectrum of tumor-reactive T cells. One explanation for how T-cells become activated against tumor antigens is by cross-presentation, during which tumor proteins are phagocytosed, digested with proteasomes, and presented via major histocompatibility complex class I to T cells for activation. Two hypothesized classes of tumor-associated proteins, defective ribosomal products (DRiPs) and short-lived proteins (SLiPs), are abundant in tumor cells, but are unstable and only transiently expressed before being degraded by tumor cell proteosomes. It has been hypothesized that these DRiP/SLiP antigens could potentially facilitate anti-tumor immune responses and could form the basis of a novel anti-tumor vaccine.

The DRibbles multi-valent vaccine is created by disrupting degradation of intracellular proteins by the ubiquitin proteasome system. It consists of autophagosome vesicles that are enriched with DRiPs and SLiPs, known tumor-associated antigens, mediators of innate immunity, and surface markers that encourage phagocytosis and cross-presentation by antigen-presenting cells. The first ‘off-the-shelf’ allogeneic human DRibbles vaccine, DPV-001, was derived from autophagosome products of two NSCLC cell lines, one of mixed histology and one from an adenocarcinoma [60]. It contains multiple TLR agonists and > 130 potential NSCLC antigens, many as prospective altered-peptide ligands. DPV-001 cancer vaccine induces and/or augments immunity against many relevant cancer antigens. In a phase II trial, patients with stage III NSCLC received cyclophosphamide induction therapy, before being randomized to DPV-001 alone, with GM-CSF or with imiquimod [61]. Patients receiving DPV-001 had a significant increase in total (CD4 and CD8) T cells over that seen with controls and the increase in CD4 T cells was similar to that seen in patients receiving ipilimumab. Vaccination induced or increased IgG antibody responses against targets over-expressed by NSCLC, correlating with activated Th1 cells in whole blood samples. New or augmented antibody responses were observed with continued vaccination [62].

Antibody responses to antigens over-expressed in NSCLC were detected as ‘waves’ with possible co-coordination of antibody and T cell responses after vaccination. T cell contraction, a natural component of a T cell response to antigen, may be responsible for these variations and raises the possibility of combining vaccination with T cell agonists that blunt contraction by augmenting T cell expansion and sustaining antigen-specific T cells. For example, OX40 ligation increases IL-2 production and IL-2R expression and enhances CD4 and CD8 T cell effector differentiation and the generation of long-lived memory cells [63]. A clinical trial is planned in patients with advanced TNBC to assess a combination strategy involving DPV-001 vaccine plus a T cell agonist with or without checkpoint blockade.

The development of personalized vaccine therapy that integrates patient-specific neoantigens into the ‘off-the-shelf’ DPV-001 vaccine is also planned. In this approach, patients can begin vaccinations with DPV-001, prior to obtaining the tumor biopsy used for neoantigen determination and personalized vaccine production.

## Key points

- A goal of treatment is to provide a large number of relevant antigens in order to activate a broad spectrum of tumor-reactive T cells.
- DRiP/SLiP antigens could potentially facilitate anti-tumor immune responses and could form the basis of a novel anti-tumor vaccine.
- DPV-001, derived from autophagosome products of two NSCLC cell lines, contains multiple TLR agonists and > 130 potential NSCLC antigens, many as prospective altered-peptide ligands and induces and/or augments immunity against many relevant cancer antigens.
- The development of personalized vaccine therapy that integrates patient-specific neoantigens into the ‘off-the-shelf’ DPV-001 vaccine is planned.

### Next target for immune checkpoint blockade in melanoma

**Fig. 2:**
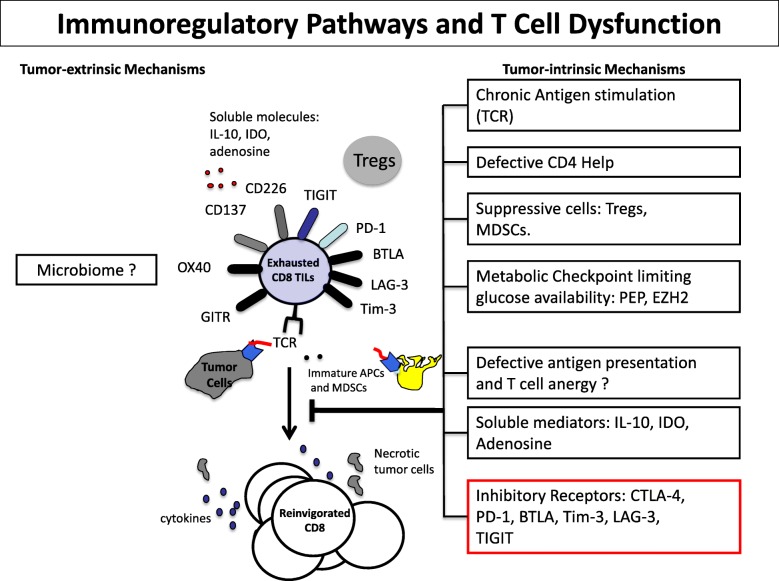
Immunoregulatory pathways in the tumor microenvironment and T cell dysfunction

Chronic antigen exposure can lead to T cell exhaustion. Exhausted T cells upregulate multiple inhibitory receptors/immune checkpoints, including PD-1, CTLA-4, TIM-3, LAG-3, and T Cell ITIM Domain (TIGIT) (Fig. 2).

These bind to their ligands that are highly expressed in the TME. There is also evidence of additive/synergistic effects on tumor antigen-specific CD8+ T cell expansion and function with dual blockade with anti-PD-1/PD-L1 antibodies together with antibodies targeting additional inhibitory receptors.

TIM-3 is a negative regulator of Th1 immune responses and spontaneous NY-ESO-1-specific CD8+ T cells as well as CD8+ TILs in solid tumors upregulate PD-1 and TIM-3. Ex vivo TIM-3 + PD1+ NY-ESO-1-specific CD8+ T cells and TILs represent a dysfunctional T cell population. TIM-3 blockade enhanced cytokine production and proliferation of NY-ESO-1-specific CD8+ T cells upon prolonged antigen stimulation is additive/synergistic with PD-1 blockade [64]. TSR-022 is a humanized anti-TIM-3 IgG4 antibody that binds to TIM-3 with high affinity and has potent in vitro and in vivo activity. In part 1 of the AMBER study, TSR-022 was dose-escalated to a 1200 mg flat dose with no dose-limiting toxicities. In part 2, TSR-022 was combined with TSR-024, an anti-PD-1 agent, in patients PD-1 refractory melanoma and NSCLC [65]. TSR-022 in combination with TSR-042 demonstrated clinical activity with objective responses in patients with post-PD-1 NSCLC and melanoma. The combination was also well tolerated with incidence of grade ≥ 3 treatment-related adverse events of 6.7%, with increased lipase and maculo-papular rash the most frequent.

The inhibitory receptor TIGIT and its competing costimulatory receptor DNAM-1/CD226 regulate innate and adaptive immune responses to tumors. TIGIT and PD-1 regulate the expansion and function of tumor antigen-specific CD8+ T cells and CD8+ TILs in melanoma patients [66]. TIGIT ligands are highly expressed in metastatic melanoma and many other solid tumors and dual TIGIT/PD-1 blockade increases the proliferation of tumor antigen-specific CD8+ T cells. TIGIT is also highly upregulated by human Tregs in the TME, whereas there is decreased expression of its competing co-stimulatory receptor CD226 [67]. In contrast to TIGIT, CD226 disrupts Treg-mediated suppression and stability in the periphery and at tumor sites. PVR-mediated activation of CD226 partially reverses TIGIT+ CD4+ Treg-induced immunosuppression and decreases Foxp3 expression in TIGIT+ CD4+ Tregs of patients with advanced melanoma. A high TIGIT/CD226 ratio in Tregs together with high PVR expression in the TME promotes Treg stability and suppressive functions. Altogether, our findings support the development of combinatorial therapies to target the TIGIT/CD226 axis in solid tumors to augment innate and adaptive immune responses to cancer.

## Key points

- TSR-022 is a humanized anti-TIM-3 IgG4 antibody that binds to TIM-3 with high affinity and has potent in vitro and in vivo activity.
- TSR-022 in combination with TSR-042 demonstrated clinical activity with objective responses in patients with post-PD-1 NSCLC and melanoma.
- The inhibitory receptor TIGIT and its competing costimulatory receptor DNAM-1/CD226 regulate innate and adaptive immune responses to tumors.
- Data support the development of combinatorial therapies to target the TIGIT/CD226 axis in solid tumors to augment innate and adaptive immune responses to cancer.

### Targeting immune escape of head and neck cancer: dangers and opportunities

The incidence of HPV+ head and neck tumors is increasing and these are typically more responsive to treatment than tobacco and alcohol-related cancers, which may in part be immune-mediated. PD-1+ CD8+ T cells with an activated phenotype may be a favourable prognostic biomarker in HPV+ patients. PD-1 expression has been shown to be upregulated on head and neck cancer (HNC) patient TILs, with a higher frequency of PD-1+ TILs in HPV+ patients [68]. Higher fractions of PD-1^low^ T cells were associated with HPV positivity and better outcome. As such, the extent of PD-1 expression on CD8+ TILs may be a potential biomarker for anti-PD-1-based immunotherapy. Total and PD-1+ NK cells are also significantly higher in the circulation of HNC patients and are associated with improved clinical outcome. These cells are also enriched in the TME. Elevated expression of NKp46 in HNC specimens (TCGA) associates with better survival and strongly correlates with PD-1 but not TIM-3 or CTLA-4 [69]. PD-1 blockade increases cetuximab-mediated NK cell activation against HNC targets with high PD-L1 expression. Therefore, blocking the PD-1/PD-L1 axis may be a useful strategy to reverse immune evasion of HNC tumors with high PD-L1 expression during cetuximab therapy by reversing NK cell dysfunction.

In the Active8 randomized clinical trial, the addition of the TLR-8 agonist motolimod to the EXTREME regimen was well tolerated but did not improve PFS or OS in the overall population [70]. However, significant benefits were observed in HPV+ patients, with significantly longer PFS and OS, as well as in patients with injection site reactions, suggesting that TLR-8 stimulation may benefit subset- and biomarker-selected patients.

The addition of nivolumab to a cetuximab-radiotherapy regimen for patients with newly diagnosed intermediate and high-risk local-regionally advanced SCCHN has been shown to be safe and feasible in the ongoing RTOG3504 trial [71]. The JAVELIN Head and Neck 100 study is a phase III randomized clinical trial assessing the efficacy of avelumab in combination with chemoradiotherapy compared with placebo in combination with chemoradiation for high-risk SCCHN, while UPCI 15–132 is assessing sequential versus concomitant pembrolizumab plus chemoradiation.

Immunotherapy is also being assessed in the neoadjuvant setting, with the CheckMate 358 trial investigating the safety and feasibility of neoadjuvant nivolumab in patients with resectable HPV+/− SCCHN. In 29 patients, nivolumab was well tolerated, with no delays to surgery due to adverse events, and resulted in tumor reductions within 1 month in nearly half of evaluable patients [72].

## Key points

- PD-1+ expression may be a favourable prognostic biomarker in HPV+ HNC patients.
- Blocking the PD-1/PD-L1 axis may be a useful strategy to reverse immune evasion of HNC tumors with high PD-L1 expression during cetuximab therapy by reversing NK cell dysfunction.
- The addition of nivolumab to a cetuximab-radiotherapy regimen for patients with newly diagnosed intermediate and high-risk local-regionally advanced SCCHN has been shown to be safe and feasible.
- The safety and feasibility of neoadjuvant nivolumab is also being evaluated in patients with resectable HPV+/− SCCHN.

### Systems immunology and tumor microenvironment

Immunophenotyping of tumors may provide prognostic information and the Immunoscore was first proposed as a potential approach for the classification of cancer in 2012. More recently, international validation has shown that it provides a reliable estimate of the risk of recurrence in patients with colon cancer and it has been proposed as a new component of a TNM-Immune classification of cancer [73].

The efficacy of immunotherapies depends on the immune contexture and the ability to unleash pre-existing immunity. Tumors can be categized on the basis of their immune status as immune-infiltrated (hot), altered (Immune-excluded or immune-suppressed) and immune desert (cold) and it is critical to understand the mechanisms responsible for each in order to boost antitumor immunity [74]..

A key question is whether there is an immune escape at the metastatic stage? In analysis of resected metastases from colorectal cancer patients, Immunoscore and T and B cell score in the least immune-infiltrated metastases were the strongest predictors for disease-free survival and OS [75]. Assessment of immune cell types of 603 whole-slide metastases and primary colorectal tumors from 222 colorectal cancer patients showed high intra-metastasis, inter-metastasis and intra-patient heterogeneity [76]. Small metastases frequently had a low Immunoscore and T and B cell score, while a high Immunoscore was associated with a lower number of metastases. The Immunoscore from a single biopsy was more reliable than PD-L1 expression as a predictor of survival.

Current theories of cancer evolution are tumor cell-centric with none involving a role of the immune system. A parallel selection model of metastatic progression, where branched evolution in space and time could be traced back to immune-escaping clones has now been proposed [77]. Multiplexed analyses reveal highly heterogeneous genomic patterns and immune cell infiltration between metastases and that clonal evolution patterns during metastatic progression depend on the immune contexture at the metastatic site. Transmission of tumor clones occurs from one metastasis to consecutive metastases with multiparallel tumor evolution and diverse tumor clones. Non-recurrent eliminated clones are immunoedited while persistent clones are immune-privileged (not immunoedited), despite the presence of TILs. Non-recurrent clones (< 4 years) have a low immunoediting score. For immunoediting to occur, a high Immunoscore is necessary but alone is not sufficient, since high-Immunoscore may not show immunoediting. Characterization of immune-privileged metastases revealed tumor-intrinsic and tumor-extrinsic mechanisms of escape, with different escape mechanisms delineated by lack of adaptive immunity or immunoediting. Immunoediting and Immunoscore are predictive factors of metastasis recurrence. Distance between CD3+ cells and Ki67+ tumor cells as well as metastasis size are also associated with metastatic dissemination. The lowest recurrence risk was associated with high Immunoscore, occurrence of immunoediting, and low tumor burden. This work represents the first demonstration in Human that tumor clone dissemination are dependent upon the immune system, and more precisely upon the immune contexture, the Immunoscore and the immunoediting [77].

Because of different escape mechanisms, there is a need for different combination therapies.

## Key points

The Immunoscore has been proposed as a new component of a TNM-Immune classification of cancer.

- Tumors can be categized on the basis of their immune status as immune-infiltrated (hot), altered (immune-excluded or immune-suppressed) and immune desert (cold) and it is critical to understand the mechanisms responsible for each in order to boost antitumor immunity.
- Analysis of resected metastases from colorectal cancer patients showed that Immunoscore and T and B cell score in the least immune-infiltrated metastases were the strongest predictors for disease-free survival and OS.
- The Immunoscore from a single biopsy may be a more reliable than PD-L1 expression as a predictor of survival.
- Immunoediting and Immunoscore are predictive factors of metastasis recurrence.

### Conclusions

Immunotherapy of cancer has made major advances in recent years and checkpoint inhibitors have become recognised as a standard of care in several different types of cancer. Increased understanding of the complex interactions between tumours and the host immune response (including the mechanistic impact of combination therapies and tumor and immune cell metabolism) and the therapeutic implications of these findings is leading to the development of novel therapeutic strategies across different cancers. In particular, research into a wide range of different and potentially synergistic immunotherapy combinations is ongoing, novel cellular therapies are being fine-tuned, and the role for vaccines is being better elucidated and will soon lead to more durable responses for higher numbers of patients.

## Abbreviations

ACT: Adoptive cell transfer
ADP: Adenosine DiPhosphate
B2M: β 2 microglobulin
BLIA: Basal-like immune-activated
BRCA: Breast Related Cancer Antigens
CAR: Chimeric antigen receptor
CTLA: Cytotoxic T-lymphocyte-associated antigen
CSF: Cerebrospinal fluid
DC: Dendritic cell
DCR: Disease control rate
DOR: Duration of response
DNAM-1: DNAX accessory molecule 1
DRiPs: Defective ribosomal products
EGFR: Epidermal growth factor receptor
EGFRvIII: Epidermal growth factor receptor variant III
EOC: Endometrial ovarian cancer
FOXP3: Forkhead box P3
GITR: Glucocorticoid-induced TNFR family related gene
GM-CSF: Granulocyte-macrophage colony-stimulating factor
GU: Genitourinary
HNC: Head and neck cancer
HPV: Human papillomavirus
HRD: Homologous recombination deficiency
ICOS: Inducible Co-Stimulator
IFN: Interferon
IgG: Immunoglobulin G
IHC: Immunohistochemistry
IL: Interleukin
IMC: Imaging mass cytometry
irRC: Immune-related response criteria
LAG-3: Lymphocyte-activating gene-3
LAR: Luminal androgen receptor
MAGE-A3: Melanoma-associated antigen 3
MCC: Merkel cell carcinoma
MMR: Mismatch repair
MSI-H: Microsatellite instability-high
MSKCC: Memorial Sloan Kettering Cancer Center
NK: Natural killer
NSCLC: Non-small-cell lung cancer
NY-ESO-1: New York esophageal squamous cell carcinoma 1
ORR: Overall response rate
OS: Overall survival
OVV: Oncolytic vaccinia virus
PARP: Poly-ADP ribose polymerase
PDAC: Pancreatic ductal adenocarcinoma
PD-1: Programmed death-1
PD-L1: Programmed death ligand-1
PCK1: Phosphoenolpyruvate carboxykinase 1
PEP: Phosphoenolpyruvate
PFS: Progression-free survival
PPAR: Peroxisome proliferator-activated receptor
RECIST: Response Evaluation Criteria in Solid Tumors
RCC: Renal-cell carcinoma
ROS: Reactive Oxygen Species
SCCHN or HNSCC: squamous-cell carcinoma of the head and neck
SLiPs: Short-lived proteins
STAT3: Signal transducer and activator of transcription 3
TCGA: The Cancer Genome Atlas
TCR: T-cell receptor
TGF: Transforming growth factor
Th: T helper
TIGIT: T cell immunoreceptor with Ig and ITIM domains
TILs: Tumor-infiltrating lymphocytes
TIM-3: T-cell immunoglobulin and mucin-domain containing-3
TLR: Toll-like receptor
TMB: Tumor mutational burden
TME: Tumor microenvironment
TNBC: Triple-negative breast cancer
TNFR: Tumor necrosis factor receptor
TNM: Tumor (lymph) node metastasis
Treg: T regulatory cell
T-VEC: Talimogene laherparepvec
VEGF: Vascular endothelial growth factor
